# Global Trends of Medical Misadventures Using International Classification of Diseases, Tenth Revision Cluster Y62-Y69 Comparing Pre–, Intra–, and Post–COVID-19 Pandemic Phases: Protocol for a Retrospective Analysis Using the TriNetX Platform

**DOI:** 10.2196/54838

**Published:** 2024-04-17

**Authors:** Rosario Caruso, Marco Di Muzio, Emanuele Di Simone, Sara Dionisi, Arianna Magon, Gianluca Conte, Alessandro Stievano, Emanuele Girani, Sara Boveri, Lorenzo Menicanti, Mary A Dolansky

**Affiliations:** 1 Health Professions Research and Development Unit IRCCS Policlinico San Donato San Donato Milanese Italy; 2 Department of Biomedical Sciences for Health University of Milan Milano Italy; 3 Department of Clinical and Molecular Medicine Sapienza University of Rome Rome Italy; 4 Nursing, Technical and Rehabilitation Department DaTeR Azienda Unità Sanitaria Locale di Bologna Bologna Italy; 5 Department of Clinical and Experimental Medicine University of Messina Messina Italy; 6 Centre of Excellence for Nursing Scholarship Rome Italy; 7 Laboratory of Biostatistics and Data Management Scientific Directorate IRCCS Policlinico San Donato San Donato Milanese Italy; 8 Scientific Directorate IRCCS Policlinico San Donato San Donato Milanese Italy; 9 Frances Payne Bolton School of Nursing and School of Medicine Case Western Reserve University Cleveland, OH United States

**Keywords:** COVID-19, curve-fitting analyses, health care quality, health care safety, International Classification of Diseases, Tenth Revision, ICD-10, incidence rates, safety, TriNetX

## Abstract

**Background:**

The COVID-19 pandemic has sharpened the focus on health care safety and quality, underscoring the importance of using standardized metrics such as the *International Classification of Diseases, Tenth Revision* (*ICD-10*). In this regard, the *ICD-10* cluster Y62-Y69 serves as a proxy assessment of safety and quality in health care systems, allowing researchers to evaluate medical misadventures. Thus far, extensive research and reports support the need for more attention to safety and quality in health care. The study aims to leverage the pandemic’s unique challenges to explore health care safety and quality trends during prepandemic, intrapandemic, and postpandemic phases, using the *ICD-10* cluster Y62-Y69 as a key tool for their evaluation.

**Objective:**

This research aims to perform a comprehensive retrospective analysis of incidence rates associated with *ICD-10* cluster Y62-Y69, capturing both linear and nonlinear trends across prepandemic, intrapandemic, and postpandemic phases over an 8-year span. Therefore, it seeks to understand how these trends inform health care safety and quality improvements, policy, and future research.

**Methods:**

This study uses the extensive data available through the TriNetX platform, using an observational, retrospective design and applying curve-fitting analyses and quadratic models to comprehend the relationships between incidence rates over an 8-year span (from 2015 to 2023). These techniques will enable the identification of nuanced trends in the data, facilitating a deeper understanding of the impacts of the COVID-19 pandemic on medical misadventures. The anticipated results aim to outline complex patterns in health care safety and quality during the COVID-19 pandemic, using global real-world data for robust and generalizable conclusions. This study will explore significant shifts in health care practices and outcomes, with a special focus on geographical variations and key clinical conditions in cardiovascular and oncological care, ensuring a comprehensive analysis of the pandemic’s impact across different regions and medical fields.

**Results:**

This study is currently in the data collection phase, with funding secured in November 2023 through the Ricerca Corrente scheme of the Italian Ministry of Health. Data collection via the TriNetX platform is anticipated to be completed in May 2024, covering an 8-year period from January 2015 to December 2023. This dataset spans pre-pandemic, intra-pandemic, and early post-pandemic phases, enabling a comprehensive analysis of trends in medical misadventures using the ICD-10 cluster Y62-Y69. The final analytics are anticipated to be completed by June 2024. The study's findings aim to provide actionable insights for enhancing healthcare safety and quality, reflecting on the pandemic's transformative impact on global healthcare systems.

**Conclusions:**

This study is anticipated to contribute significantly to health care safety and quality literature. It will provide actionable insights for health care professionals, policy makers, and researchers. It will highlight critical areas for intervention and funding to enhance health care safety and quality globally by examining the incidence rates of medical misadventures before, during, and after the pandemic. In addition, the use of global real-world data enhances the study’s strength by providing a practical view of health care safety and quality, paving the way for initiatives that are informed by data and tailored to specific contexts worldwide. This approach ensures the findings are applicable and actionable across different health care settings, contributing significantly to the global understanding and improvement of health care safety and quality.

**International Registered Report Identifier (IRRID):**

PRR1-10.2196/54838

## Introduction

A wealth of scholarly research and empirical data underscores the imperative of prioritizing safety and quality in health care [1,2]. Seminal studies in the last 3 decades, such as the Canadian Adverse Events Study and the Harvard Medical Practice Study, have highlighted the rates of adverse events in health care settings, which led to severe, sometimes fatal, consequences for patients [3,4]. Moreover, research conducted in various international contexts has emphasized the necessity of rigorous safety protocols and the urgent need for continuous quality improvement plans [5-7]. The criticality of safety and quality in health care is not merely a theoretical construct but is supported by extensive research, governmental reports, and real-world implications [8]. Ensuring that safety and quality are at the forefront of health care delivery is pivotal for enhancing patient outcomes, reducing costs, and optimizing the effectiveness and efficiency of health care systems globally [9].

The COVID-19 pandemic has served as a watershed moment in the global health care landscape, profoundly influencing safety culture and catalyzing a renewed emphasis on quality improvement plans [10]. The unprecedented strain on health care systems worldwide has necessitated rapid adaptations in safety protocols, resource allocation, and patient care strategies. The pandemic has exposed vulnerabilities in existing health care infrastructures, compelling institutions to reevaluate and fortify their safety measures [11]. For instance, the critical importance of infection control has been magnified, leading to more stringent guidelines for personal protective equipment use, sanitation, and patient isolation [12]. Moreover, the pandemic has accelerated the adoption of telemedicine, which presents its own set of quality and safety considerations. The COVID-19 pandemic has also underscored the importance of data-driven approaches to safety and quality, as health care providers increasingly rely on real-time analytics to make informed decisions in a rapidly changing environment and use data to monitor improvements [13]. In essence, the COVID-19 pandemic has acted as a catalyst for a paradigm shift in health care safety culture, making the continuous improvement of quality not just an institutional goal but a global imperative.

In this evolving landscape of health care safety and quality improvement, the potential use of the *International Classification of Diseases, Tenth Revision* (*ICD-10*) has gained significant attention [14]. *ICD-10* serves as a standardized coding system for diagnosing a wide array of medical conditions, thereby facilitating precise communication among health care providers and enabling robust data collection for research and policy development [15]. This standardization has further enabled the use of expansive data sets for retrospective analyses, thereby contributing to targeted quality improvement initiatives. For instance, platforms such as TriNetX leverage the *ICD-10* coding system to facilitate data-driven decision-making, offering health care institutions invaluable insights into areas requiring intervention or optimization [16]. Consequently, the *ICD-10* framework stands as a pivotal instrument in elevating the culture of safety and the data needed to guide quality improvement plans, particularly in the intricate health care landscape shaped by the ongoing COVID-19 pandemic.

Within the framework of *ICD-10*, the cluster Y62-Y69, designated for “Misadventures to patients during surgical and medical care,” serves as a critical proxy for assessing patient safety and quality of care [17]. This particular cluster comprises an array of diagnostic codes that encapsulate a diverse spectrum of medical misadventures, ranging from lapses in sterile precautions to inaccuracies in dosage administration and contamination of medical or biological substances. Using these nuanced codes enables health care practitioners and academic researchers to undertake focused analyses to augment the standard of medical care. Critically, this categorization facilitates pinpointing potential vulnerabilities in existing safety protocols, thus providing empirically based insights that can be harnessed for the advancement of quality improvement strategies to improve safety.

In light of the urgent need to enhance safety and quality in health care, a comprehensive analysis focusing on the *ICD-10* cluster Y62-Y69 could offer invaluable insights. This research protocol articulates a methodical framework for examining longitudinal trends in the incidence rates associated with this specific cluster. Using the TriNetX platform, the study will encompass an 8-year period and use curve-fitting analyses. The choice of an 8-year time frame for this study serves multiple analytical purposes. First, it provides a sufficient number of data points to establish a robust trendline prior to the onset of the COVID-19 pandemic. These baseline data are crucial for understanding the pre-existing patterns and vulnerabilities in health care safety and quality, as captured by the *ICD-10* cluster Y62-Y69. Second, including data during the pandemic allows for an in-depth examination of how the health care systems adapted their safety protocols and quality improvement plans in response to the unprecedented challenges posed by COVID-19. Finally, extending the study into the postpandemic context offers a timely opportunity to assess the current state of health care safety and quality, including any lasting impacts or improvements catalyzed by the pandemic experience. Therefore, the 8-year span is methodologically sound and contextually relevant, enabling a comprehensive analysis that covers prepandemic, pandemic, and postpandemic phases.

This study aims to describe not only the linear trajectories of these rates but also the dynamics of their rate of change over time. This analytical focus has particular relevance in the context of health care systems’ responses to the COVID-19 pandemic, a transformative event that has indelibly impacted global health care. The protocol specifies the analytical methodologies to be deployed, identifies the data repositories for consultation, and outlines the statistical models for rigorous data interpretation. Ultimately, this research protocol aims to provide a robust analytical framework capable of generating empirically substantiated findings regarding the past and current state of safety. The findings are designed to inform targeted interventions for the advancement of health care safety and quality.

## Methods

### Study Design

This study will adopt an observational, retrospective design, adhering to the STROBE (Strengthening the Reporting of Observational Studies in Epidemiology) guidelines. This is a big data study, leveraging the extensive and diverse data sets available within the TriNetX platform to provide a comprehensive analysis. The Gantt Chart for research activities presents a 6-month project timeline, where the initial 3 months are allocated to retrieving data. This is followed by a period of analysis spanning 2 months. Concurrently, starting in the third month and continuing through to the sixth month, a total of 3 months is devoted to meta-elaboration and scientific writing.

### Setting and Data Source

Data will be extracted from the TriNetX platform, a global health research network that provides real-time access to clinical data. The platform’s big data capabilities enable the analysis of large, complex data sets, thereby enhancing the robustness and generalizability of the study findings.

This retrospective analysis will be done using TriNetX, a global health research network providing a deidentified data set of electronic medical records regarding demographics, diagnoses, procedures, medications, laboratory values, genomics, and visits. This network comprises routinely collected, aggregated clinical data from around 130 million patients attending 107 health care institutions in 16 countries, with data spanning from 2008 to 2023.

Data include both inpatient and outpatient care. Clinical information is collected using widespread standard terminologies, such as Systematized Nomenclature of Medicine, Logical Observation Identifiers Names and Codes, and *ICD-10*. TriNetX is certified to the International Organization for Standardization 27001:2013 standard and maintains an Information Security Management System to ensure the protection of the health care data it has access to and to meet the requirements of the Health Insurance Portability and Accountability Act (HIPAA) Security Rule.

The cohort for the study includes all patients aged 0 to 89 years old who underwent any *ICD-10-Clinical Modification* diagnosis during the period of observation. The *ICD-10-Clinical Modification* cluster Y62-Y69, which represents “Misadventures to patients during surgical and medical care,” has been identified as the outcome of the analysis. The analysis of the incidence rate of the outcome in the years between 2016 and 2023 (4 years before and 4 years after the pandemic) will be conducted globally and for specific geographic areas: North America (United States); Latin America; Europe, Middle East, and Africa; and Asia-Pacific. This analysis will use the corresponding collaborative networks within TriNetX. TriNetX offers natural language processing capabilities, which use machine learning technology from Averbis (located in Freiburg in Breisgau, Germany) to search free text from clinical charts and other records specifically for the geographical areas in the United States.

The analysis will be applied to an oncological cohort and one with cardiovascular diseases to validate the results obtained in the initial phase of the research. The oncological cohort was determined using any *ICD-10* codes related to neoplasms (C00-D49). In contrast, the cardiovascular cohort was identified using codes related to Diseases of the Circulatory System (I00-I99).

The findings will be reported according to the STROBE guideline. One of the primary goals of STROBE is to ensure a clear and transparent account of the reporting of methods and results.

### Study Period

The analysis will encompass 8 years of data, now divided into 3 distinct phases to align with the key periods of the COVID-19 pandemic: from 2018 to 2019 (prepandemic phase), from 2020 to May 2023 (intrapandemic phase, aligning with the federal COVID-19 public health emergency declaration period), and from June 2023 to the end of 2023 (early postpandemic phase). This division allows for a precise examination of health care safety and quality trends during the prepandemic phase, the active pandemic phase as defined by the federal public health emergency declaration, and the immediate aftermath of the pandemic. This approach aims to provide a more detailed and contextually relevant understanding of the varying impacts of the COVID-19 pandemic, thus offering a comprehensive view of the health care landscape and its adaptation during these critical periods.

### Variables of Interest

This study’s primary variables of interest are the incidence rates associated with the *ICD-10* cluster Y62-Y69, a specific set of diagnostic codes designated for capturing “Misadventures to patients during surgical and medical care” [17]. This cluster is of particular importance as it serves as a critical proxy for assessing the safety and quality of health care delivery. It encompasses a wide range of medical misadventures, including but not limited to lapses in sterile precautions, inaccuracies in dosage administration, and contamination of medical or biological substances. Analyzing the incidence rates will enable a comprehensive evaluation of the health care system’s performance in minimizing medical misadventures and informing targeted interventions for quality improvement and safety enhancement [18]. The limitations of these data are the self-reporting nature of documenting the “misadventures,” with the potential for underreporting.

The incidence rate, which measures the rate of new or first-time cases, is calculated using a time-sensitive approach. The denominator for the incidence rate is the product of the number of patients in the incidence proportion denominator and the number of days covered by the time interval. This ensures that the incidence rate provides a dynamic view of how safety and quality are evolving over time. Importantly, the study also incorporates a “lookback period” to exclude patients who have experienced the event of interest prior to the study period, thereby focusing only on new cases.

Incidence rates are subject to stringent criteria, including demographic matching and time window overlaps, to ensure that the data are accurate and meaningful for targeted interventions. Furthermore, the study acknowledges the potential impact of date shifting by health care organizations to protect patient health information and takes this into consideration in the analysis.

### Statistical Analysis

The primary aim of the forthcoming statistical analysis will be to decipher the linear and nonlinear trajectories of incidence rates, with a particular focus on understanding their rate of change over time. Special attention will be dedicated to evaluating the impact of the COVID-19 pandemic on these health care metrics.

The linchpin of the analytical strategy will be the application of curve-fitting analyses [19]. This advanced technique will enable researchers to construct models that elucidate the intricate relationships between the rates and the temporal variables. Specifically, polynomial regression models, including quadratic models, will be used to capture the complexity of the data trends. Quadratic models will be particularly useful for capturing nonlinear trends in the data. These models will be formulated based on the equation *y* = *ax*^2^ + *bx* + *c*, where *y* represents the incidence rate; *x* represents time; and *a*, *b*, and *c* are coefficients to be estimated. The quadratic term *ax*^2^ will allow us to understand the curvature in the data, providing insights into acceleration or deceleration trends over time. Model diagnostics, such as residual analysis and goodness-of-fit tests, will be conducted to ensure the appropriateness of the quadratic models.

Initially, curve-fitting analyses will be executed on an overall sample characterized by its extensive demographic and clinical diversity. Given the inherent heterogeneity of this sample, a rigorous validation process will be indispensable for confirming the generalizability and applicability of the observed trends.

A series of subgroup analyses will be undertaken to augment the robustness of the findings. These analyses will be stratified by various factors, including but not limited to geographic location. Data for these subgroup analyses will be sourced from the TriNetX network, which amalgamates health care data from a multitude of geographic regions. This approach will facilitate an assessment of the consistency of the observed trends across diverse subpopulations to bolster the external validity of the findings. Further stratification will be conducted based on prevalent epidemiological conditions in the fields of oncology and cardiovascular diseases. Within these conditions, specific groups identified with inclusion and exclusion criteria will be selected as they possess well-documented epidemiological profiles, serving as robust benchmarks for validation. Reperforming the curve-fitting analyses within these disease-specific cohorts will aim to corroborate the trends discerned in the overall sample.

### Ethical Considerations

#### Human Subject Research Ethics Review and Approvals

The study uses data from the TriNetX network, which adheres to international and national data protection and privacy laws, including the HIPAA in the United States and the General Data Protection Regulation in the European Union. These compliance standards ensure that the data are reliably sourced and managed ethically. Each health care organization participating in the TriNetX network is committed to strict ethical standards, including obtaining informed consent for primary data collection with provisions for secondary analysis. Therefore, the study protocol has been approved by the institutional review board of Policlinico San Donato within the Ricerca Corrente funding scheme, affirming adherence to ethical standards and contribution to health care research. No external ethical committee review was deemed necessary due to the aggregated nature of the information, emphasizing the study’s compliance with privacy and data protection principles inherent in its observational and secondary data analysis approach.

#### Informed Consent for Secondary Analysis

The study involves secondary analysis of anonymized data sets obtained from organizations within the TriNetX network. It is important to note that the original informed consent obtained by these organizations (or their respective institutional review boards) allows for secondary analysis without requiring additional consent. This approach ensures that the use of the data respects and upholds the original consent and ethical approvals.

#### Privacy and Confidentiality Protection

The data included in the study, derived from the TriNetX platform, are aggregated to ensure that individual patient data are not directly used. This aggregation process, in conjunction with the initial ethical compliance by each participating health care organization, guarantees the protection of privacy and confidentiality. The data used in the research are anonymous and deidentified, following strict data protection principles.

#### Compensation

Compensation is not applicable in the study as it revolves around the secondary analysis of existing anonymized data sets. There is no direct participant interaction, so compensation for participants is not applicable.

#### Image and Supplementary Material Identification

No individual participants or user identification is possible in any images included in the manuscript or supplementary materials. The study design protects the privacy and anonymity of all individuals involved.

### Anticipated Findings

The anticipated results of this comprehensive analysis focusing on the *ICD-10* cluster Y62-Y69 are expected to provide valuable insights into the safety and quality of health care delivery over the study period, spanning prepandemic, intrapandemic, and early postpandemic phases.

Regarding the incidence rates associated with the *ICD-10* cluster Y62-Y69, we anticipate that our analysis will reveal trends and patterns that can be attributed to various factors, including changes in safety protocols, resource allocation, and health care practices during the COVID-19 pandemic. Given the unprecedented challenges and adaptations required by health care systems worldwide, it is reasonable to expect that the pandemic may have had a significant impact on these rates.

In the prepandemic phase, our analysis aims to establish a baseline for the incidence rates, offering insights into the existing vulnerabilities in health care safety and quality captured by the *ICD-10* cluster. This baseline data will be crucial for understanding the context in which subsequent changes occurred.

During the intrapandemic phase, we anticipate observing fluctuations and shifts in the incidence rates. These changes may be indicative of the health care system’s responses to the challenges posed by the pandemic, including heightened infection control measures, changes in patient care protocols, and resource allocation adjustments. The pandemic’s impact on safety and quality may be reflected in the data.

In the early postpandemic phase, we expect to see how health care systems have adapted and evolved in response to the pandemic’s challenges. It is possible that some changes implemented during the pandemic may persist or lead to improvements in safety and quality.

Furthermore, our analysis will include stratified subgroup analyses, which may reveal variations in trends across different geographic regions and within specific disease cohorts (oncology and cardiovascular diseases). These subgroup analyses will help identify whether the observed trends are consistent across diverse populations and clinical contexts. This study aims to provide a nuanced understanding of how the COVID-19 pandemic has influenced safety and quality in health care, as reflected in the incidence rates of the *ICD-10* cluster Y62-Y69. These findings will inform targeted interventions and quality improvement strategies, contributing to the ongoing efforts to enhance health care safety and quality in a rapidly evolving health care landscape.

## Results

Our research, supported by the Ricerca Corrente funding from the Italian Ministry of Health to IRCCS Policlinico San Donato (funds secured in November 2023), is currently advancing through the data collection stage. The compilation of data, facilitated by the global health research network TriNetX, is set to encapsulate a comprehensive 8-year timeframe extending from January 2015 to December 2023. This carefully curated dataset documents the healthcare landscape across three critical phases: before the COVID-19 pandemic (pre-pandemic), during the height of the pandemic (intra-pandemic), and the period following the emergency phase of the pandemic (early post-pandemic). Such temporal delineation is pivotal for our investigation into the trends and fluctuations in medical misadventures classified under the ICD-10 cluster Y62-Y69, offering a lens through which to view the nuances of healthcare safety and quality across different global healthcare contexts.

With data collection anticipated to be completed by May 2024, and final analysis expected by June 2024, our study is on track to fulfill its objective of dissecting the intricate patterns of medical misadventures during a period marked by unprecedented global health challenges. The results are anticipated to equip healthcare professionals, policymakers, and stakeholders with the insights necessary to fortify healthcare systems against future adversities, thereby enhancing the overall safety and quality of care delivered to patients worldwide

## Discussion

### Principal Findings

The primary expectation of this study is to elucidate the linear and nonlinear trajectories of incidence rates associated with the *ICD-10* cluster Y62-Y69 over time. Through the application of curve-fitting analyses, specifically quadratic models, we anticipate revealing intricate patterns in the data that may not be discernible through simpler linear models.

In the context of the COVID-19 pandemic, we expect to observe significant fluctuations in these rates, potentially manifesting as spikes or declines corresponding to various phases of the pandemic. These observations will be critical for understanding the pandemic’s impact on health care safety and quality, particularly in medical misadventures. Furthermore, examining the postpandemic response to these rates will be of particular interest. These results will provide insights into the resilience and adaptability of health care systems in returning to prepandemic safety and quality levels or possibly achieving even better standards.

Upon conducting subgroup analyses, we expect that the trends observed in the overall sample will be validated in specific subpopulations. These subpopulations will be stratified by geographic location and specific epidemiological conditions within oncology and cardiovascular diseases. The validation of trends across these diverse subpopulations will lend greater credibility and generalizability to our findings.

Moreover, we anticipate that our stringent data selection criteria for validating the overall models will yield accurate and meaningful results. These results are expected to inform targeted interventions to improve the safety and quality of health care delivery, fulfilling the study’s ultimate objective.

However, it is essential to acknowledge the limitations that may arise from the study. These could include potential biases in the original data documentation and collection and the challenges associated with interpreting complex statistical models. One specific limitation is the retrospective nature of the study, which may introduce recall bias and limit the ability to establish causal relationships. Retrospective studies often rely on existing records and data, which may not have been collected for research purposes, affecting the data’s quality and completeness. In addition, despite the application of data selection criteria, the possibility of unmeasured or residual confounding factors cannot be entirely excluded. These factors might influence the observed associations and outcomes. This limitation is characteristic of retrospective studies and should be considered when interpreting our findings. Furthermore, we acknowledge the challenges associated with analyzing data from broad and heterogeneous geographic areas such as Europe, the Middle East, Africa, and Asia-Pacific. The diversity in health care systems, practices, and patient populations across these regions may limit the uniformity and specific applicability of our findings. This variation is important when interpreting our results and their implications in these diverse settings. We recognize this as a limitation of our study and suggest caution in generalizing the findings uniformly across these broad geographic areas.

Despite these limitations, the strength of this study lies in its use of global real-world big data. The TriNetX network provides a rich data set that captures a broad range of demographic and clinical variables, enhancing the study’s generalizability and applicability. The use of real-world data allows for a contemporary understanding of medical misadventures, as it reflects on the complexities and variabilities inherent in everyday health care settings. This aspect is a significant advantage over controlled clinical trials, which often operate under idealized conditions that may not be representative of the real world.

Future research will focus on addressing these limitations and possibly using other advanced statistical techniques for a more in-depth analysis. For instance, machine learning algorithms could be used to identify hidden patterns and relationships in the data, providing a more comprehensive understanding of the factors influencing medical misadventures. In line with our future research directions, we aim to delve deeper into specific indicators like complications, side effects, mortality, and morbidity in subsequent studies. These indicators, when analyzed in homogeneous subgroups of patients with and without the presence of the *ICD-10* cluster Y62-Y69, could provide critical insights into the impact of medical misadventures on patient outcomes. This focus will augment our current understanding of relevant safety-related patterns and guide targeted interventions to mitigate such occurrences in health care settings.

### Conclusions

The upcoming study is poised to offer a multifaceted exploration of the incidence rates associated with medical misadventures, as defined by the *ICD-10* cluster Y62-Y69. The research is designed to use advanced statistical models, such as quadratic equations, to capture both linear and nonlinear trends over time. This approach will be particularly illuminating in the context of the pre–, intra–, and post–COVID-19 pandemic analyses, a period in history that has introduced unique challenges and disruptions to health care systems globally. Global real-world data stand as a significant strength, offering a more pragmatic view of health care safety and quality that can typically be achieved through controlled clinical trials or other primary studies. The study’s findings are anticipated to be of considerable value to health care professionals, policy makers, and researchers alike. The results will not only shed light on the current state of medical misadventures but also provide actionable insights for targeted interventions aimed at improving health care safety and quality.

## Appendix

Multimedia Appendix 1 Peer-review report from the independent reviewers of the “Ricerca Corrente” fund of IRCCS Policlinico San Donato (Italy) and previous version of the protocol.

## Abbreviations

ICD-10: International Classification of Diseases, Tenth Revision
HIPAA: Health Insurance Portability and Accountability Act
STROBE: Strengthening the Reporting of Observational Studies in Epidemiology

## Data Availability

The data sets generated and analyzed during this study are available from the corresponding author on reasonable request.
