# Supplementary material for: Global Trends of Medical Misadventures Using International Classification of Diseases, Tenth Revision Cluster Y62-Y69 Comparing Pre–, Intra–, and Post–COVID-19 Pandemic Phases: Protocol for a Retrospective Analysis Using the TriNetX Platform
Source: JMIR Res Protoc. 2024 Apr 17;13:e54838. doi: 10.2196/54838 (PMC11025602; doi:10.2196/54838)
Supplement: Multimedia Appendix 1 [file resprot_v13i1e54838_app1.pdf]

Project Evaluation Grid for Funding Application (Ricerca Corrente), max 5.000 €

Peer Reviewer:_______Dr. Marco Ranucci___________

Scoring Guide:

1 = Poor
2 = Fair
3 = Good
4 = Very Good
5 = Excellent

Project summary and budget justification:

Title: Retrospective Analysis of ICD-10 Cluster Y62-Y69 within TriNetX: A Protocol for Evaluating Healthcare Safety and Quality across Pre, Intra, and Post-COVID-19 Pandemic Phases

Duration: 6 months

Total Budget Request: maximum €5.000

Budget Breakdown:

- **Article APC (Article Processing Charges):** These are fees required by open-access journals to cover the entire editorial process, from submission to publication. Given the importance and timeliness of our results, ensuring that our research is freely available to the global community is strategic.
- **Dissemination Activities:** Sharing our findings is not limited to academic publications. We intend to engage in various activities, including webinars, conferences, and outreach programs, to ensure that our insights reach a broad spectrum of stakeholders, from practitioners to policymakers.

Rationale: The requested budget primarily centers on ensuring that our findings are extensively disseminated. Given the pandemic's global impact and the crucial nature of healthcare safety and quality, we believe that broadly communicating the emerging results from this project will significantly benefit the healthcare community.

Criteria	Description	Score (1-5)	Comments
**Relevance & Justification**	How well does the proposal align with the fund's objectives?	4	There is a good alignment
**Background**	Is there a clear understanding of existing literature and the problem's significance?	4	Yes
**Objectives**	Are the study objectives well-defined and relevant?	3	There are well defined, but I'm not sure that they are clinically relevant
**Methodology**			
**- Study Design**	Is the study design appropriate for the objectives? Does it follow STROBE guidelines?	4	Yes, it is
**- Population/Setting**	Is the chosen population and setting relevant and adequately described?	3	Oncologic and cardiovascular PTS are included. Given the mission of our Institution, I would concentrate on cardiovascular.

**- Variables**	Are the variables well-defined, relevant, and based on existing knowledge?	**4**	Yes
**- Data Sources/Measurement**	Are the data sources reliable? Is the measurement methodology clear and valid?	**4**	The platform TriNetX seems to be adequate.
**- Bias**	Are potential biases acknowledged and addressed?	**3**	It is a retrospective study with all the potential biases involved.
**- Statistical Methods**	Are the statistical methods appropriate and well-explained?	**4**	Yes
**Expertise & Ethics**		**2**	
**Impact & Deliverables**	What are the expected outcomes? How will they contribute to the field or benefit society?		I do not think there is a real outcome. It is more a description of the state of the art in the setting of safety in medical procedures, within a time frame inclusive of COVID pandemic.

**Ethical Considerations**	Are ethical aspects considered and appropriately addressed?	**5**	Yes
**Other**		**4**	Globally, It is an original study.

Total score (a minimum score of 40/60 is required to obtain the fund): 44

Final comment: The requested amount is limited and adequate to the study needs.

Project Evaluation Grid for Funding Application (Ricerca Corrente), max 5.000 €

Peer Reviewer:______Prof. Massimo Piepoli_________

Scoring Guide:

1 = Poor
2 = Fair
3 = Good
4 = Very Good
5 = Excellent

Project summary and budget justification:

Title: Retrospective Analysis of ICD-10 Cluster Y62-Y69 within TriNetX: A Protocol for Evaluating Healthcare Safety and Quality across Pre, Intra, and Post-COVID-19 Pandemic Phases

Duration: 6 months

Total Budget Request: maximum €5.000

Budget Breakdown:

- **Article APC (Article Processing Charges):** These are fees required by open-access journals to cover the entire editorial process, from submission to publication. Given the importance and timeliness of our results, ensuring that our research is freely available to the global community is strategic.
- **Dissemination Activities:** Sharing our findings is not limited to academic publications. We intend to engage in various activities, including webinars, conferences, and outreach programs, to ensure that our insights reach a broad spectrum of stakeholders, from practitioners to policymakers.

Rationale: The requested budget primarily centers on ensuring that our findings are extensively disseminated. Given the pandemic's global impact and the crucial nature of healthcare safety and quality, we believe that broadly communicating the emerging results from this project will significantly benefit the healthcare community.

Criteria	Description	Score (1-5)	Comments
**Relevance & Justification**	How well does the proposal align with the fund's objectives?	**5**	Importance of the topic: critical evaluation of the performance of the health systems in a stress situation like the pandemia
**Background**	Is there a clear understanding of existing literature and the problem's significance?	**5**	
**Objectives**	Are the study objectives well-defined and relevant?	**5**	Clearly presented and discussed
**Methodology**			
**- Study Design**	Is the study design appropriate for the objectives? Does it follow STROBE guidelines?	**4**	Retrospective analysis: confounders cannot be excluded. This should be included in the limitation paragraph
**- Population/Setting**	Is the chosen population and setting relevant and adequately described?	**4**	Some of the geographic area considered in the analysis are very broad and not uniform [in particular Europe, Middle East, and Africa (EMEA), and Asia-Pacific (APAC)] to draw any strong and useful conclusion: this should be added in the limitation section

**- Variables**	Are the variables well-defined, relevant, and based on existing knowledge?	**4**	1. A more detailed list of indicators that are going to be evaluated is missing: complications, side effects, mortality and morbidity, hospitalization<br>2. Is it possible to perform an analysis for specific cardiovascular condition, such as AMI/CAD, HF, SCD, myocarditis)
**- Data Sources/Measurement**	Are the data sources reliable? Is the measurement methodology clear and valid?	**4**	
**- Bias**	Are potential biases acknowledged and addressed?	**4**	Retrospective analysis: confounders cannot be excluded. This should be included in the limitation paragraph
**- Statistical Methods**	Are the statistical methods appropriate and well-explained?	**4**	References supporting the complex statistical analysis are not clearly included
**Expertise & Ethics**			
**Impact & Deliverables**	What are the expected outcomes? How will they contribute to the field or benefit society?	**5**	Importance of the topic: critical evaluation of the performance of the health systems in a stress situation like the pandemia

**Ethical Considerations**	Are ethical aspects considered and appropriately addressed?		Not applicable: retrospective analysis
**Other**			

Total score (a minimum score of 40/60 is required to obtain the fund): 44/60

Final comment:

Aim: to conduct a longitudinal analysis of the incidence rates associated with the ICD-10 cluster Y62-Y69. The study seeks to capture both linear and non-linear trends over an eight-year period, encompassing the pre-pandemic, intra-pandemic, and post-pandemic phases.

Strengths:

1. Importance of the topic: critical evaluation of the performance of the health systems in a stress situation like the pandemia
2. Appropriate selection of indicators of performance (cluster Y62-69)
3. Appropriate identification of the most important clinical conditions to be investigated, ie oncological cohort and a cardiovascular diseases cohort.
4. Complex analytical evaluation and statistical analysis

Observations

Major points

3. **Study design** Retrospective analysis: confounders cannot be excluded. This should be included in the limitation paragraph
    a. **RESPONSE: We agree that retrospective analyses, by their nature, face challenges in completely accounting for all confounding variables. We have amended the limitations section of our manuscript to explicitly state this inherent challenge.**
4. **Variables of interest** A more detailed list of indicators that are going to be evaluated is missing: complications, side effects, mortality and morbidity, hospitalization
    a. **RESPONSE: We recognize the importance of indicators such as complications, side effects, mortality, and morbidity, and their relevance in the context of healthcare safety and quality. However, in this initial epidemiological step of**

our analytics, we have not included the further exploration of these additional indicators. This decision was made to focus on the foundational aspects of the ICD-10 cluster Y62-Y69 analysis. To address your concern, we have added a sentence in the discussion section of our manuscript. This addition emphasizes that future projects should explore these indicators in more depth, particularly in homogeneous subgroups of patients showing the presence of the cluster and those without the cluster. This approach will indeed add value by providing a comprehensive understanding of the importance of examining these aspects in the context of medical misadventures. We fully agree that such an analysis would be instrumental in enhancing the understanding of healthcare safety and quality, and we appreciate your suggestion for improving the scope and depth of our research.

5. Is it possible to perform an analysis for specific cardiovascular condition, such as AMI/CAD, HF, SCD, myocarditis)
    a. **RESPONSE: We acknowledge the potential value of such detailed analyses in understanding the nuances of medical misadventures in different cardiovascular conditions. However, the current phase of our study is focused on a broader epidemiological exploration using the ICD-10 cluster Y62-Y69. This initial phase aims to establish foundational insights into the general trends and patterns of medical misadventures. In response to your suggestion, we have included a sentence in the discussion section of our manuscript. This addition highlights our intention to conduct more in-depth analyses in future research phases. Such analyses would be invaluable in dissecting the intricacies of medical misadventures within specific cardiovascular conditions.**
6. **Study Period.** If the aim is comparing the pre-pandemic, intra-pandemic, and post-pandemic phases, why the study period of 8 years has been divided in only 2 parts (2016-19 and 2020-23) and not in 3 parts (2018-19; 20-21; and 22-23) in order to differentiate between the pre-, intra-, and post-pandemic phases
    a. **RESPONSE: We appreciate your input regarding the division of the study period. In light of your suggestion and considering the end of the federal COVID-19 PHE declaration on May 11, 2023, we have revised the study period in our manuscript.**
7. **Setting and data source** Some of the geographic area considered in the analysis are very broad and not uniform [in particular Europe, Middle East, and Africa (EMEA), and Asia-Pacific (APAC)] to draw any strong and useful conclusion: this should be added in the limitation section
    a. **RESPONSE: In response to your comment, we have added a statement in the limitations section of our manuscript. This addition acknowledges the challenge posed by the broad and heterogeneous nature of these geographic areas in our analysis.**
8. **Statistical analysis** equation $y = ax^2 + bx + c$. How was it selected?

Minor points

- References are not included in the text.
- **RESPONSE: fixed using the JMIR Research Protocol style**

- A central figure listing the main steps of the study (ie data collection, analysis and output) is missing.

- **RESPONSE: done.**

Protocol 1.2

Retrospective Analysis of ICD-10 Cluster Y62-Y69 within TriNetX: A Protocol for Evaluating Healthcare Safety and Quality across Pre, Intra, and Post-COVID-19 Pandemic Phases

Abstract

Background: The criticality of healthcare safety and quality has been underscored by extensive scholarly research and governmental and agency reports, also being further magnified by the challenges posed by the COVID-19 pandemic. The International Classification of Diseases, Tenth Revision (ICD-10), and specifically the cluster Y62-Y69, serves as a pivotal tool for evaluating these aspects in healthcare settings.
Objective: The primary aim of this study is to conduct a longitudinal analysis of the incidence rates associated with the ICD-10 cluster Y62-Y69. The study seeks to capture both linear and non-linear trends over an eight-year period, encompassing the pre-pandemic, intra-pandemic, and post-pandemic phases.
Methods: Utilizing an observational, retrospective design, the study will leverage big data from the TriNetX platform. Advanced statistical techniques, including curve-fitting analyses and quadratic models, will be employed to decipher the intricacies of the incidence rates over time.
Results: While the study is yet to be conducted, it is anticipated to reveal complex patterns in healthcare safety and quality, in the context of the COVID-19 pandemic. The use of global real-world data is expected to enhance the robustness and generalizability of the findings.
Conclusions: The study aims to offer a comprehensive exploration of healthcare safety and quality trends as defined by the ICD-10 cluster Y62-Y69. The anticipated findings are expected to serve as a valuable resource for healthcare professionals, policymakers, and researchers, providing actionable insights for targeted interventions and funding mechanisms to improve healthcare safety and quality.

Keywords: COVID-19; Curve-fitting analyses; Healthcare quality; Healthcare safety; ICD-10; Longitudinal analysis; Incidence rates; Safety; TriNetX

Introduction

A wealth of scholarly research and empirical data underscores the imperative of prioritizing safety and quality in healthcare [1,2]. Seminal studies in the last three decades, such as the Canadian Adverse Events Study and the Harvard Medical Practice Study, have highlighted the rates of adverse events in healthcare settings, which led to severe, sometimes fatal, consequences for patients [3,4]. Moreover, research conducted in various international contexts has emphasized the necessity of rigorous safety protocols and the urgent need for continuous quality improvement plans [5–7]. The criticality of safety and quality in healthcare is not merely a theoretical construct but is supported by extensive research, governmental reports, and real-world implications [8]. Ensuring that safety and quality are at the forefront of healthcare delivery is pivotal for enhancing patient outcomes, reducing costs, and optimizing the effectiveness and efficiency of healthcare systems globally [9].

The COVID-19 pandemic has served as a watershed moment in the global healthcare landscape, profoundly influencing safety culture and catalyzing a renewed emphasis on quality improvement plans [10]. The unprecedented strain on healthcare systems worldwide has necessitated rapid adaptations in safety protocols, resource allocation, and patient care strategies. The pandemic has exposed vulnerabilities in existing healthcare infrastructures, compelling institutions to reevaluate and fortify their safety measures [11]. For instance, the critical importance of infection control has been magnified, leading to more stringent guidelines for personal protective equipment use, sanitation, and patient isolation [12]. Moreover, the pandemic has accelerated the adoption of telemedicine, which presents its own set of quality and safety considerations. The COVID-19 pandemic has also underscored the importance of data-driven approaches to safety and quality, as healthcare providers increasingly rely on real-time analytics to make informed decisions in a rapidly changing environment and use data to monitor improvements [13]. In essence, the COVID-19 pandemic has acted as a catalyst for a paradigm shift in healthcare safety culture, making the continuous improvement of quality not just an institutional goal but a global imperative.

In this evolving landscape of healthcare safety and quality improvement, the potential utility of the International Classification of Diseases, Tenth Revision (ICD-10), has gained significant attention [14]. ICD-10 serves as a standardized coding system for diagnosing a wide array of medical conditions, thereby facilitating precise communication among healthcare providers and enabling robust data collection for research and policy development [15]. This standardization has further enabled the utilization of expansive datasets for retrospective analyses, thereby contributing to targeted quality improvement initiatives. For instance, platforms such as TriNetX leverage the ICD-10 coding system to facilitate data-driven decision-making, offering healthcare institutions invaluable insights into areas requiring intervention or optimization [16]. Consequently, the ICD-10 framework stands as a pivotal instrument in elevating the culture of safety and the data needed to guide quality improvement plans, particularly in the intricate healthcare landscape shaped by the ongoing COVID-19 pandemic.

Within the framework of ICD-10, the cluster Y62-Y69, designated for "Misadventures to patients during surgical and medical care," serves as a critical proxy for assessing patient safety and quality of care [17]. This particular cluster comprises an array of diagnostic codes that encapsulate a diverse spectrum of medical misadventures, ranging from lapses in sterile precautions to inaccuracies in dosage administration and contamination of medical or

biological substances. Utilizing these nuanced codes enables healthcare practitioners and academic researchers to undertake focused analyses with the objective of augmenting the standard of medical care. Critically, this categorization facilitates pinpointing potential vulnerabilities in existing safety protocols, thus providing empirically-based insights that can be harnessed for the advancement of quality improvement strategies to improve safety.

In light of the urgent need to enhance safety and quality in healthcare, a comprehensive analysis focusing on the ICD-10 cluster Y62-Y69 could offer invaluable insights. This research protocol articulates a methodical framework for examining longitudinal trends in the incidence rates associated with this specific cluster. Utilizing the TriNetX platform, the study will encompass an eight-year period and employ curve-fitting analyses. The choice of an eight-year timeframe for this study serves multiple analytical purposes. Firstly, it provides a sufficient number of data points to establish a robust trendline prior to the onset of the COVID-19 pandemic. This baseline data is crucial for understanding the pre-existing patterns and vulnerabilities in healthcare safety and quality, as captured by the ICD-10 cluster Y62-Y69. Secondly, including data during the pandemic allows for an in-depth examination of how the healthcare systems adapted their safety protocols and quality improvement plans in response to the unprecedented challenges posed by COVID-19. Lastly, extending the study into the post-pandemic context offers a timely opportunity to assess the current state of healthcare safety and quality, including any lasting impacts or improvements catalyzed by the pandemic experience. Therefore, the eight-year span is methodologically sound and contextually relevant, enabling a comprehensive analysis that covers pre-pandemic, pandemic, and post-pandemic phases.

The aim of this study is to describe not only the linear trajectories of these rates but also the dynamics of their rate of change over time. This analytical focus has particular relevance in the context of healthcare systems' responses to the COVID-19 pandemic, a transformative event that has indelibly impacted global healthcare. The protocol specifies the analytical methodologies to be deployed, identifies the data repositories for consultation, and outlines the statistical models for rigorous data interpretation. Ultimately, this research protocol aims to provide a robust analytical framework capable of generating empirically substantiated findings regarding the past and current state of safety. The findings are designed to inform targeted interventions for the advancement of healthcare safety and quality.

Methods

Study design

This study will adopt an observational, retrospective design, adhering to the Strengthening the Reporting of Observational Studies in Epidemiology (STROBE) guidelines. This is a big data study, leveraging the extensive and diverse datasets available within the TriNetX platform to provide a comprehensive analysis. Figure 1 depicts the timeline of the study.

Setting and data source

Data will be extracted from the TriNetX platform, a global health research network that provides real-time access to clinical data. The platform's big data capabilities enable the analysis of large, complex datasets, thereby enhancing the robustness and generalizability of the study findings.

This retrospective analysis will be done using TriNetX, a global health research network providing a de-identified data set of electronic medical records regarding demographics, diagnoses, procedures, medications, laboratory values, genomics and visits. This network comprises clinical routinely-collected, aggregated data from around 130 million patients attending 107 healthcare institutions in 16 countries, with data spanning 2008-2023.

Data include both inpatient and outpatient care. Clinical information are collected using widespread standard terminologies, such as Systematized Nomenclature of Medicine (SNOMED), Logical Observation Identifiers Names and Codes (LOINC), and ICD-10. TriNetX is certified to the International Organization for Standardization (ISO) 27001:2013 standard and maintains an Information Security Management System to ensure the protection of the healthcare data it has access to and to meet the requirements of the Health Insurance Portability and Accountability Act Security Rule.

The cohort for the study includes all patients aged 0 to 89 years old who underwent any ICD-10-Clinical Modification (ICD-10-CM) diagnosis during the period of observation. The ICD-10-CM cluster Y62-Y69, which represents "Misadventures to patients during surgical and medical care", has been identified as the outcome for the analysis. The analysis of the incidence rate of the outcome in the years between 2016 and 2023 (4 years before and 4 years after the pandemic) will be conducted globally and for specific geographic areas: North America (US), Latin America (LATAM), Europe, Middle East, and Africa (EMEA), and Asia-Pacific (APAC). This analysis will utilize the corresponding collaborative networks within TriNetX. TriNetX offers natural language processing capabilities, which utilize machine learning technology from Averbis (located in Freiburg im Breisgau, DE) to search free text from clinical charts and other records specifically for the US geographic area.

The analysis will be applied to an oncological cohort and a cardiovascular diseases cohort as a means of validating the results obtained in the initial phase of the research. The oncological cohort was determined using any ICD-10 codes related to Neoplasms (C00-D49). In contrast, the cardiovascular cohort was identified using codes related to Diseases of the Circulatory System (I00-I99).

Findings will be reported according to the Strengthening the Reporting of Observational Studies in Epidemiology (STROBE) guideline. One of the primary goals of STROBE is to ensure a clear and transparent account of the reporting of methods and results.

Study Period

The analysis will encompass eight years of data, now divided into three distinct phases to align with the key periods of the COVID-19 pandemic: 2018-2019 (pre-pandemic), 2020-May 2023 (intra-pandemic, aligning with the federal COVID-19 PHE declaration period), and June 2023-2023 end (early post-pandemic). This division allows for a precise examination of healthcare safety and quality trends during the pre-pandemic, the active pandemic phase as defined by the federal PHE declaration, and the immediate aftermath of the pandemic. This approach aims to provide a more detailed and contextually relevant understanding of the varying impacts of the COVID-19 pandemic, thus offering a comprehensive view of the healthcare landscape and its adaptation during these critical periods.

Variables of interest

This study's primary variables of interest are the incidence rates associated with the ICD-10 cluster Y62-Y69, a specific set of diagnostic codes designated for capturing

"Misadventures to patients during surgical and medical care" [17]. This cluster is of particular importance as it serves as a critical proxy for assessing the safety and quality of healthcare delivery. It encompasses a wide range of medical misadventures, including but not limited to lapses in sterile precautions, inaccuracies in dosage administration, and contamination of medical or biological substances. Analyzing incidence rates will enable a comprehensive evaluation of the healthcare system's performance in minimizing medical misadventures, thereby informing targeted interventions for quality improvement and safety enhancement [18]. The limitations of this data are the self-report nature of documenting the "misadventures" with the potential for underreporting.

The incidence rate, which measures the rate of new or first-time cases, is calculated using a time-sensitive approach. The denominator for the incidence rate is the product of the number of patients in the incidence proportion denominator and the number of days covered by the time interval. This ensures that the incidence rate provides a dynamic view of how safety and quality are evolving over time. Importantly, the study also incorporates a "lookback period" to exclude patients who have experienced the event of interest prior to the study period, thereby focusing only on new cases.

Incidence rates are subject to stringent criteria, including demographic matching and time window overlaps, to ensure that the data are accurate and meaningful for targeted interventions. Furthermore, the study acknowledges the potential impact of date shifting by healthcare organizations to protect patient health information and takes this into consideration in the analysis.

Statistical analysis

The primary aim of the forthcoming statistical analysis will be to decipher the linear and non-linear trajectories of incidence rates, with a particular focus on understanding their rate of change over time. Special attention will be dedicated to evaluating the impact of the COVID-19 pandemic on these healthcare metrics.

The linchpin of the analytical strategy will be the application of curve-fitting analyses [19]. This advanced technique will enable researchers to construct models that elucidate the intricate relationships between the rates and temporal variables. Specifically, polynomial regression models, including quadratic models, will be employed to capture the complexity of the data's trends. Quadratic models will be particularly useful for capturing non-linear trends in the data. These models will be formulated based on the equation $y = ax^2 + bx + c$, where y represents the incidence rate, x represents time, and a, b, and c are coefficients to be estimated. The quadratic term ax^2 will allow us to understand the curvature in the data, providing insights into acceleration or deceleration trends over time. Model diagnostics, such as residual analysis and goodness-of-fit tests, will be conducted to ensure the appropriateness of the quadratic models.

Initially, curve-fitting analyses will be executed on an overall sample characterized by its extensive demographic and clinical diversity. Given the inherent heterogeneity of this sample, a rigorous validation process will be indispensable for confirming the generalizability and applicability of the observed trends.

A series of subgroup analyses will be undertaken to augment the robustness of the findings. These analyses will be stratified by various factors, including but not limited to geographic location. Data for these subgroup analyses will be sourced from the TriNetX network, which amalgamates healthcare data from a multitude of geographic regions. This

approach will facilitate an assessment of the consistency of the observed trends across diverse subpopulations to bolster the external validity of the findings. Further stratification will be conducted based on prevalent epidemiological conditions in the fields of oncology and cardiovascular diseases. Within these conditions, specific groups identified with inclusion/exclusion criteria will be selected as they possess well-documented epidemiological profiles, serving as robust benchmarks for validation. Re-performing the curve-fitting analyses within these disease-specific cohorts will aim to corroborate the trends discerned in the overall sample.

Ethical considerations

The study leverages data from the TriNetX network, which is compliant with international and national data protection and privacy laws, including the Health Insurance Portability and Accountability Act (HIPAA) in the United States and the General Data Protection Regulation (GDPR) in the European Union. This compliance ensures that the data are reliable, ethically sourced, and managed properly. All data utilized in this research are derived from anonymized sources and available in an aggregate manner.

Discussion

The primary expectation of this study is to elucidate the linear and non-linear trajectories of incidence rates associated with the ICD-10 cluster Y62-Y69 over time. Through the application of curve-fitting analyses, specifically quadratic models, we anticipate revealing intricate patterns in the data that may not be discernible through simpler linear models.

In the context of the COVID-19 pandemic, we expect to observe significant fluctuations in these rates, potentially manifesting as spikes or declines corresponding to various phases of the pandemic. These observations will be critical for understanding the pandemic's impact on healthcare safety and quality, particularly in the realm of medical misadventures. Furthermore, examining the post-pandemic response to these rates will be of particular interest. These results will provide insights into the resilience and adaptability of healthcare systems in returning to pre-pandemic safety and quality levels or possibly achieving even better standards.

Upon conducting subgroup analyses, we expect that the trends observed in the overall sample will be validated in specific subpopulations. These subpopulations will be stratified by geographic location and specific epidemiological conditions within oncology and cardiovascular diseases. The validation of trends across these diverse subpopulations will lend greater credibility and generalizability to our findings.

Moreover, we anticipate that our stringent data selection criteria for validating the overall models will yield accurate and meaningful results. These results are expected to inform targeted interventions to improve the safety and quality of healthcare delivery, fulfilling the study's ultimate objective.

However, it is essential to acknowledge the limitations that may arise from the study. These could include potential biases in the original data documentation and collection and the challenges associated with interpreting complex statistical models. One specific limitation is the retrospective nature of the study, which may introduce recall bias and limit the ability to establish causal relationships. Retrospective studies often rely on existing records and data, which may not have been collected for research purposes, thereby affecting the quality and

completeness of the data. In addition, despite the application of data selection criteria, the possibility of unmeasured or residual confounding factors cannot be entirely excluded. These factors might influence the observed associations and outcomes. This limitation is characteristic of retrospective studies and should be considered when interpreting our findings. Furthermore, we acknowledge the challenges associated with analyzing data from broad and heterogeneous geographic areas such as Europe, Middle East, Africa, and Asia-Pacific. The diversity in healthcare systems, practices, and patient populations across these regions may limit the uniformity and specific applicability of our findings. This variation is an important consideration when interpreting our results and their implications in these diverse settings. We recognize this as a limitation of our study and suggest caution in generalizing the findings uniformly across these broad geographic areas.

Despite these limitations, the strength of this study lies in its use of global real-world big data. The TriNetX network provides a rich dataset that captures a broad range of demographic and clinical variables, enhancing the study's generalizability and applicability. The use of real-world data allows for a contemporary understanding of medical misadventures, as it reflects the complexities and variabilities inherent in everyday healthcare settings. This aspect is a significant advantage over controlled clinical trials, which often operate under idealized conditions that may not be representative of the real world.

Future research will focus on addressing these limitations and possibly employing other advanced statistical techniques for a more in-depth analysis. For instance, machine learning algorithms could be used to identify hidden patterns and relationships in the data, providing a more comprehensive understanding of the factors influencing medical misadventures. In line with our future research directions, we aim to delve deeper into specific indicators like complications, side effects, mortality, and morbidity in subsequent studies. These indicators, when analyzed in homogeneous subgroups of patients with and without the presence of the ICD-10 cluster Y62-Y69, could provide critical insights into the impact of medical misadventures on patient outcomes. This focus will augment our current understanding on relevant safety-related patterns and guide targeted interventions to mitigate such occurrences in healthcare settings.

Conclusions

The upcoming study is poised to offer a multi-faceted exploration of the incidence rates associated with medical misadventures, as defined by the ICD-10 cluster Y62-Y69. The research is designed to employ advanced statistical models, such as quadratic equations, to capture both linear and non-linear trends over time. This approach will be particularly illuminating in the context of the pre, intra, and post-COVID-19 pandemic analyses, a period in history which has introduced unique challenges and disruptions to healthcare systems globally. The use of global real-world data stands as a significant strength, offering a more pragmatic view of healthcare safety and quality than can typically be achieved through controlled clinical trials. The study's findings are anticipated to be of considerable value to healthcare professionals, policymakers, and researchers alike. The results will not only shed light on the current state of medical misadventures but also provide actionable insights for targeted interventions aimed at improving healthcare safety and quality.

Acknowledgements

This study was partially supported by Ricerca Corrente funding from Italian Ministry of Health to IRCCS Policlinico San Donato.

Conflicts of Interest

None declared.

Abbreviations

ICD-10: International Classification of Diseases, Tenth Revision
Y62-Y69: ICD-10 cluster for "Misadventures to patients during surgical and medical care"
STROBE: Strengthening the Reporting of Observational Studies in Epidemiology
TriNetX: Global health research network platform
SNOMED: Systematized Nomenclature of Medicine
LOINC: Logical Observation Identifiers Names and Codes
ISO 27001:2013: International Organization for Standardization standard for information security management
ISMS: Information Security Management System
HIPAA: Health Insurance Portability and Accountability Act
ICD-10-CM: International Classification of Diseases, Tenth Revision, Clinical Modification
US: United States
LATAM: Latin America
EMEA: Europe, Middle East, and Africa
APAC: Asia-Pacific
Averbis: Machine learning technology for natural language processing

References

1. Jha AK, Prasopa-Plaizier N, Larizgoitia I, Bates DW, On Behalf of the Research Priority Setting Working Group of the WHO World Alliance for Patient Safety. Patient safety research: an overview of the global evidence. Quality and Safety in Health Care 2010 Feb 1;19(1):42–47. doi: 10.1136/qshc.2008.029165

2. Institute of Medicine (US) Committee on Quality of Health Care in America. Crossing the Quality Chasm: A New Health System for the 21st Century. Washington (DC): National Academies Press (US); 2001. PMID:25057539ISBN:978-0-309-07280-9

3. Brennan TA, Leape LL, Laird NM, Hebert L, Localio AR, Lawthers AG, Newhouse JP, Weiler PC, Hiatt HH. Incidence of Adverse Events and Negligence in Hospitalized Patients: Results of the Harvard Medical Practice Study I. N Engl J Med 1991 Feb 7;324(6):370–376. doi: 10.1056/NEJM199102073240604

4. Baker GR. The Canadian Adverse Events Study: the incidence of adverse events among hospital patients in Canada. Canadian Medical Association Journal 2004 May 25;170(11):1678–1686. doi: 10.1503/cmaj.1040498

5. Alanazi NH, Falqi TA. Healthcare Managers' Perception on Patient Safety Culture. Glob J Qual Saf Healthc 2023 Feb;6(1):6–14. PMID:37260855

6. Jha AK, Larizgoitia I, Audera-Lopez C, Prasopa-Plaizier N, Waters H, Bates DW. The global burden of unsafe medical care: analytic modelling of observational studies. BMJ Qual Saf 2013 Oct;22(10):809–815. PMID:24048616

7. Giannetta N, Katigri MR, Azadboni TT, Caruso R, Liquori G, Dionisi S, De Leo A, Di Simone E, Rocco G, Stievano A, Orsi GB, Napoli C, Di Muzio M. Knowledge, Attitude, and Behaviour with Regard to Medication Errors in Intravenous Therapy: A Cross-Cultural Pilot Study. Healthcare 2023 Feb 3;11(3):436. doi: 10.3390/healthcare11030436

8. Azyabi A, Karwowski W, Davahli MR. Assessing Patient Safety Culture in Hospital Settings. Int J Environ Res Public Health 2021 Mar 3;18(5):2466. PMID:33802265

9. Kilcullen MP, Bisbey TM, Ottosen MJ, Tsao K, Salas E, Thomas EJ. The Safer Culture Framework: An Application to Healthcare Based on a Multi-Industry Review of Safety Culture Literature. Hum Factors 2022 Feb;64(1):207–227. doi: 10.1177/00187208211060891

10. Brborović O, Brborović H, Hrain L. The COVID-19 Pandemic Crisis and Patient Safety Culture: A Mixed-Method Study. IJERPH 2022 Feb 16;19(4):2237. doi: 10.3390/ijerph19042237

11. Magon A, Caruso R. Addressing a potential crisis in the Italian National Health System. The Lancet 2023 Apr;401(10384):1262–1263. doi: 10.1016/S0140-6736(23)00450-6

12. Waters R. What has the pandemic taught us about our infection control measures? BDJ In Pract 2021 May;34(5):18–19. doi: 10.1038/s41404-021-0763-y

13. Zhang Q, Gao J, Wu JT, Cao Z, Dajun Zeng D. Data science approaches to confronting the COVID-19 pandemic: a narrative review. Phil Trans R Soc A 2022 Jan 10;380(2214):20210127. doi: 10.1098/rsta.2021.0127

14. Otero Varela L, Doktorchik C, Wiebe N, Quan H, Eastwood C. Exploring the differences in ICD and hospital morbidity data collection features across countries: an international survey. BMC Health Serv Res 2021 Dec;21(1):308. doi: 10.1186/s12913-021-06302-w

15. Jetté N, Quan H, Hemmelgarn B, Drosler S, Maass C, Moskal L, Paoin W, Sundararajan V, Gao S, Jakob R, Ustün B, Ghali WA, IMECCHI Investigators. The development, evolution, and modifications of ICD-10: challenges to the international comparability of morbidity data. Med Care 2010 Dec;48(12):1105–1110. PMID:20978452

16. Wang W, Wang C-Y, Wang S-I, Wei JC-C. Long-term cardiovascular outcomes in COVID-19 survivors among non-vaccinated population: A retrospective cohort study from the TriNetX US collaborative networks. EClinicalMedicine 2022 Nov;53:101619. PMID:35971425

17. ICD10data.com. 2023 ICD-10-CM Codes Y62-Y69: Misadventures to patients during surgical and medical care. 2022. Available from: https://www.icd10data.com/ICD10CM/Codes/V00-Y99/Y62-Y69 [accessed Sep 7, 2023]

18. Spronk I, Korevaar JC, Poos R, Davids R, Hilderink H, Schellevis FG, Verheij RA, Nielen MMJ. Calculating incidence rates and prevalence proportions: not as simple as it seems. BMC Public Health 2019 Dec;19(1):512. doi: 10.1186/s12889-019-6820-3

19. Arlinghaus S. Practical Handbook of Curve Fitting. 1st ed. Boca Raton: CRC Press; 2023. doi: 10.1201/9781003418221ISBN:978-1-00-341822-1
